# Evaluation of Three Amorphous Drug Delivery Technologies to Improve the Oral Absorption of Flubendazole

**DOI:** 10.1016/j.xphs.2016.03.003

**Published:** 2016-09

**Authors:** Monica Vialpando, Stefanie Smulders, Scott Bone, Casey Jager, David Vodak, Michiel Van Speybroeck, Loes Verheyen, Katrien Backx, Peter Boeykens, Marcus E. Brewster, Jens Ceulemans, Hector Novoa de Armas, Katrien Van Geel, Emma Kesselaers, Vera Hillewaert, Sophie Lachau-Durand, Greet Meurs, Petros Psathas, Ben Van Hove, Geert Verreck, Marieke Voets, Ilse Weuts, Claire Mackie

**Affiliations:** 1 Johnson and Johnson, Pharmaceutical Research and Development, Division of Janssen Pharmaceutica, Beerse, Belgium; 2 Bend Research, Bend, Oregon 97701; 3 Formac Pharmaceuticals, Leuven, Belgium

**Keywords:** amorphous, poorly water soluble drugs, formulation, solid dispersion, flubendazole, spray drying, ordered mesoporous silica, oral absorption, filarial disease, ACN, acetonitrile, API, active pharmaceutical ingredient, AUC, area under the curve, *C*_max_, maximum drug concentration, DCM, dichloromethane, FA, formic acid, FeSSIF, fed state simulated intestinal fluid, HPMC, hydroxypropylmethylcellulose, HPMCAS, hydroxypropylmethylcellulose acetate succinate, MPSDD, modified process spray-dried dispersion, NH_4_Ac, ammonium acetate, OMS, ordered mesoporous silica, *P*_app_, apparent permeability coefficient, PK, pharmacokinetic, PVP VA, polyvinylpyrrolidone-vinyl acetate, RH, relative humidity, RS, reference solution, SDD, spray-dried dispersion, SSDD, standard spray-dried dispersion, *T*_g_, glass transition temperature, *T*_m_, melting temperature, TPGS, tocopheryl polyethylene glycol succinate

## Abstract

This study investigates 3 amorphous technologies to improve the dissolution rate and oral bioavailability of flubendazole (FLU). The selected approaches are (1) a standard spray-dried dispersion with hydroxypropylmethylcellulose (HPMC) E5 or polyvinylpyrrolidone-vinyl acetate 64, both with Vitamin E d-α-tocopheryl polyethylene glycol succinate; (2) a modified process spray-dried dispersion (MPSDD) with either HPMC E3 or hydroxypropylmethylcellulose acetate succinate (HPMCAS-M); and (3) confining FLU in ordered mesoporous silica (OMS). The physicochemical stability and *in vitro* release of optimized formulations were evaluated following 2 weeks of open conditions at 25°C/60% relative humidity (RH) and 40°C/75% RH. All formulations remained amorphous at 25°C/60% RH. Only the MPSDD formulation containing HPMCAS-M and 3/7 (wt./wt.) FLU/OMS did not crystallize following 40°C/75% RH exposure. The OMS and MPSDD formulations contained the lowest and highest amount of hydrolyzed degradant, respectively. All formulations were dosed to rats at 20 mg/kg in suspension. One FLU/OMS formulation was also dosed as a capsule blend. Plasma concentration profiles were determined following a single dose. *In vivo* findings show that the OMS capsule and suspension resulted in the overall highest area under the curve and *C*_max_ values, respectively. These results cross-evaluate various amorphous formulations and provide a link to enhanced biopharmaceutical performance.

## Introduction

Studies indicate that drug candidates are becoming increasingly difficult to formulate as a function of 3 confluent trends: (1) the use of high throughput screening to identify drug leads; (2) the nature of drug candidate isolation from chemical processes, which biases systems to complex forms; and (3) the nature of contemporary drug targets, which often diverge from the chemical space that is known to provide useful oral bioavailabilities.^1^, ^2^ Although conventional formulation strategies are initially sought based on their lower developmental risk and cost, often these approaches do not provide for adequate exposure in preclinical and clinical assessments. There is a strong industrial sensitivity toward aggregating the risk of new drug delivery systems. Therefore, the aim of this study was to evaluate the biopharmaceutical performance of 2 emerging drug delivery technologies with a more conventional approach as a performance-based drug development risk assessment with flubendazole (FLU) as the model compound.

FLU belongs to the group of benzimidazole carbamates and was first marketed as Fluvermal^®^ by Janssen Pharmaceutica in the mid-1970s as an anthelminthic agent against gastrointestinal parasites. In an early 1980s study in Mexico, FLU demonstrated superior activity compared with diethylcarbamazine against the filarial parasite, *Onchocerca volvulus*, after 12 months of follow-up.^3^ Although this study had some limitations (i.e., the total exposure from 5 weekly injections was not assessed), the activity of FLU was supported by the absence of recurrent dermal microfilaria, a surrogate marker for living worms capable of reproduction. It was recently estimated that 26 and 129 million people (mainly in sub-Saharan Africa) are infected with the filarial diseases onchocerciasis and lymphatic filariasis, respectively.^4^, ^5^

As Fluvermal was originally designed to treat gastrointestinal parasites, systemic uptake was not required. Also, it is well known that orally administered methylcarbamate benzimidazole results in poor systemic exposure in most species.^6^ Therefore, the first step was to re-formulate FLU to improve the dissolution rate, solubility, and therefore the systemic exposure, which is necessary to target the filarial larvae and adult worm. Here, we evaluate 3 amorphous drug delivery technologies to achieve this.

Solid dispersions were first defined in 1971 as one or more active ingredients in an inert carrier or matrix in the solid state.^7^ Due to advances in manufacturing process technologies, solid dispersions are now routinely produced by spray drying as a means to enhance the dissolution rate and solubility of poorly soluble compounds.^8^ Here, the resulting active pharmaceutical ingredient (API) is molecularly dispersed within the polymeric carrier matrix.^8^, ^9^, ^10^ Following exposure to aqueous media, the API is released in its supersaturated state as individual molecules and/or fine colloidal particles and the polymeric carrier impedes precipitation, leading to its enhanced performance.^9^, ^11^, ^12^ Examples of marketed solid dispersions include Sporanox^®^ and Kaletra^®^.

One limitation of this technique is that both the API and polymer must be soluble in the liquid phase. Because this solvent must easily evaporate for particle formulation to occur, a low vapor pressure is also necessary. For compounds with low solubility in solvent systems that meet the previously mentioned criteria, or to avoid non-compendial solvents, heat can be used to increase the solubility. The level of heat that is required for total solubilization depends on many factors. For compounds with solubility limitations similar to FLU, an in-line heat exchanger can be used to heat the sample above the boiling point of the solvent. The in-line heat exchanger technology was chosen based on the very limited exposure (<30 s) that is required to completely dissolve the API. Using heat to increase solubility in volatile organic solvents has associated risks. Particle size and morphology of the ingoing API can have an effect on the dissolution kinetics during heating and can require longer residence times in the heat exchanger. Chemical stability during heating is also a consideration. However, due to the limited time the spray solution is exposed to the elevated temperature, thermal chemical degradation is typically not observed. Once the API has been dissolved in the heat exchanger, a specialized atomizer (termed a flash nozzle) is used. This nozzle is unique to the heating process based on atomization taking place by flash boiling of the solvent.^13^, ^14^

Adsorption onto ordered mesoporous silica (OMS) is another example of a new enabling technology that improves the performance of poorly soluble compounds by improving their dissolution rate and solubility and thereby enhancing oral bioavailability.^15^, ^16^, ^17^ It is increasingly attracting the attention of industrial scientists due to several factors such as its burgeoned interest in the academic world.^18^, ^19^ Their cylindrical and uniform-sized pore structure serves as the key attribute to improve the dissolution rate of poorly soluble compounds. A concentrated drug solution is loaded into the pores through capillary forces. The dissolved API is added in cycles to allow solvent evaporation, leading to a confined amorphous API.^20^ When the mesopore size is only a few times larger than the drug molecule, the confined API is unable to crystallize, thus exhibiting a higher free energy and consequently higher solubility when compared to its crystalline counterpart.^21^, ^22^

Formulations from each drug delivery technology were screened using a variety of solid-state characterization tools and *in vitro* dissolution experiments in biorelevant media. The physical and chemical stability of the formulations following 2 weeks of open storage at 25°C/60% RH and 40°C/75% RH was also assessed. Finally, the 2 lead formulations from each amorphous technology were selected to evaluate systemic exposure in rats. The results from this study provide further insight into industrial formulation considerations of these emerging technologies while linking them to their *in vivo* performance.

## Materials and Methods

### Powder Manufacturing

#### Standard Spray-Dried Dispersions

The feedstock solution was prepared by dissolving either a 1/9/0.5 or 1/3/0.15 weight ratio of FLU (Shaanxi Hanjiang Pharmaceutical Group, Hanzhong City, China)/polymer/Vitamin E d-α-tocopheryl polyethylene glycol 1000 succinate (TPGS; Barentz NV, Zaventem, Belgium) in 1/9 (wt./wt.) 98%-100% formic acid (FA; Merck, Overijse, Belgium)/dichloromethane (DCM; Merck). The selected polymers were either hydroxypropylmethylcellulose (HPMC E5; Dow Chemical, Terneuzen, The Netherlands) or polyvinylpyrrolidone-vinyl acetate 64 (PVP VA 64; Kollidon^®^64, BASF, Ludwigshaven, Germany). A Büchi 290 (Flawil, Switzerland) equipped with an inert loop was used to spray dry in closed loop conditions with an inlet and outlet temperature of 65°C and 45°C, respectively, and a spray rate of 8 g/min under nitrogen flow. The damp powder was removed from the collector and dried ≤20 h in a vacuum oven (Heraeus, Liederkerke, Belgium) set to 45°C and 200 mbar under nitrogen flow. To investigate the influence of drying time, the 1/3/0.15, FLU/HPMC E5/Vit. E TPGS was also dried for 5 days under the same conditions.

#### Modified Process Spray-Dried Dispersions

The stock suspension was prepared by suspending FLU and polymer in the appropriate solvent systems at the various ratios of FLU/polymer. Formulations containing hydroxypropylmethylcellulose acetate succinate-M (HPMCAS-M; Shin-Etsu, Tokyo, Japan) were suspended in 9:1 acetone:water (Honeywell Burdick & Jackson, Muskegon, MI) and formulations containing HPMC E3 (Dow Chemical, Midland, MI) were suspended in 9:1 methanol:water (Honeywell Burdick & Jackson). The approach for ensuring complete dissolution of the API in this spray-drying process is 2-fold. First, the solubility for the given solvent system and temperature is previously determined prior to the modified spray-drying process. To ensure dissolution, a process temperature that is slightly higher than the temperature at which the solubility was observed is selected. Second, the lack of crystalline material present in the final dispersion is evidence that complete dissolution of the API was achieved in the heat exchanger. The material was prepared by spray drying using a modified spray dryer similar in scale to a ProCepT (Zalzate, Belgium). The spray-drying equipment was operated in open loop with the temperature of the heat exchanger operating at 120°C-130°C. These formulations were spray dried at a solvent flow rate of 25 g/min with an outlet temperature of 45°C-55°C. The wet MPSDD (modified process spray-dried dispersion) powder was placed in a convection tray dryer at 40°C and ambient humidity for 19.5 h.

#### Ordered Mesoporous Silica

OMS synthesis was based on the procedure described by Jammaer et al.^23^ Two solvent systems were used to load FLU into OMS containing 7-nm diameter pores using the incipient wetness impregnation method.^20^ A target drug load of 2/8 (wt./wt.) FLU/OMS was prepared using a 100 mg/mL solution of FLU in 1/1 (vol./vol.) FA (Chem-Lab, Zedelgem, Belgium)/DCM (Fisher Scientific, Aalst, Belgium).

Pure FA was used to dissolve 200 mg/mL FLU in order to achieve 3/7, 4/6, and 1/1 (wt./wt.) FLU/OMS. The damp material was post dried in a Binder VD53 vacuum oven (Tuttlingen, Germany) set to 40°C at a reduced pressure of 50 mbar for ≤20 h.

### *In Vitro* Characterization

#### Powder X-Ray Diffraction

The solid-state nature of FLU was determined by powder X-ray diffraction (PXRD) in reflection mode using an X’PertPRO diffractometer (PANalytical, Eindhoven, The Netherlands) equipped with an X’Celerator detector and spinner stage. Samples were flattened onto zero background plate holders and measured in ambient conditions by scanning from 3° to 50° 2*θ* with a 0.0167° 2*θ* step size every 60 s in reflection mode with a 1 s spinner revolution time. CuKα radiation (1.5406 Å) was used with a generator voltage and current of 45 kV and 40 mA, respectively. Diffraction patterns were analyzed using the X’Pert High Score Plus version 2.2a (PANalytical) software.

#### Modulated Differential Scanning Calorimetry

MPSDD was evaluated by modulated differential scanning calorimetry (mDSC) to determine the thermal characteristics of the formulations. These formulations were evaluated on TA Q1000 mDSC (New Castle, DE) with sub-ambient cooling system and TA Universal Analysis software. Samples were prepared as 5-mm compacts and equilibrated overnight at <5% RH. Samples were analyzed using method parameters of −20°C to 180°C at a rate of 2.5°C/min with a modulation of ±1.5°C/min.

#### *In Vitro* Dissolution

All *in vitro* dissolution tests were evaluated as suspensions for ease of comparison to the *in vivo* results (Section 2.3). Prior to dissolution testing, formulations equivalent to 50 mg API were prepared as a suspension in 0.5 wt.% Methocel (DOW Europe GmbH, Antwerpen, Belgium) containing a 2 mg API/mL concentration. Suspensions were prepared immediately prior to dissolution by first weighing an amount of formulation equal to the dose of 50 mg of API into a 20-mL syringe. The 4/6 FLU/OMS formulation was also tested as a capsule blend, which contained 33% FLU, 49% OMS, 8% sodium lauryl sulfate (BASF), and 10% Ac-Di-Sol^®^ (FMC, Cork, Ireland) weight ratio. The blend was homogenized using a mortar and pestle and placed into a size 000 HPMC capsule (Capsugel, Bornem, Belgium). The dissolution test was performed using a paddle apparatus USP type 2 (Erweka DT 12, Hausenstamm, Germany) at 75 rpm. A 2-phase biorelevant media simulating the human fed condition was selected to evaluate the *in vitro* release performance. The first phase consisted of 300 mL of 0.05-M sodium phosphate buffer pH 4.9 containing 0.2% of sodium chloride. After 60 min, a second phase of 600 mL of concentrated fed state simulated intestinal fluid (FeSSIF) was added to the vessel and stirred for an additional 2 h. Samples were collected at pre-determined time points in each phase and immediately filtered. The samples were diluted 1:9 in 1/1 (vol./vol.) N,N-dimethylformamide (Sigma-Aldrich)/water prior to analysis using ultra performance liquid chromatography (UPLC).

Microcentrifuge dissolution tests were conducted as a screening tool for the MPSDD formulations only. This test measures the supersaturation of drug above the API crystalline solubility when dosed as loose powder or a suspension into biorelevant media. The drug concentrations measured in this test were a composite of MPSDD free drug, drug in micelles, and drug suspended in solution as drug/polymer colloids. The gastric-transfer microcentrifuge dissolution test assesses the impact of dissolution after exposure to a low-pH environment. During the test, samples are transferred from gastric buffer [0.01N HCl, pH 2.0 (theoretical *C*_max_ = 1000 μgA/mL)] to simulated intestinal fluid [FaSSIF, pH 6.5 (theoretical *C*_max_ = 500 μgA/mL)]. An ultracentrifuge sample was also taken at either 90 min or 1200 min to measure the concentration of free drug plus drug in micelles.

#### Scanning Electron Microscopy

MPSDD was analyzed for particle morphology by a Hitachi Model S-3400N Scanning Electron Microscope (Krefeld, Germany). Samples were placed on a new aluminum post using adhesive tabs and were lightly spread. Care was taken to ensure the particles were not crushed or altered during sample preparation. Samples were sputter coated in a Hummer 6.2 sputter coater with AuPd target. MPSDD was then imaged at multiple magnifications to assess surface morphology.

#### Chromatography

##### High Performance Liquid Chromatography

Assay and chemical purity were quantified using a high performance liquid chromatography (HPLC) system (Waters Alliance 2695, Zellik, Belgium) consisting of a quaternary pump with gradient portion valve equipped with a 100 μL loop. The W2996 PDA detector was set to 248 nm. The mobile phase consisted of (A) 25 mM ammonium acetate (NH_4_Ac; Sigma-Aldrich) adjusted to pH 9 with ammonium hydroxide (Sigma-Aldrich) and (B) acetonitrile (ACN; Merck). Gradient elution at a constant flow rate of 1 mL/min was performed as follows: 70% of (A) decreased linearly to 66% in 2 min, followed by another linear decrease to 60% at 14 min and held constant for 1 min. Following 15 min, (A) increased linearly to 70% and held constant until 20 min. A 5-μL injection volume flowed through an X-Bridge C-18, 150 × 4.6-mm column (Waters) set to a temperature of 30°C. Reference solutions were injected *n* ≤ 5 and were linear over the concentration range of 0.05-0.5 mg/mL. Samples were injected as *n* = 1. Peaks were integrated using the Empower 3 software.

The suspensions prepared for *in vivo* dosing were also evaluated for concentration and homogeneity using HPLC-UV (Shimadzu lC20AD), consisting of a pump, oven, degasser, and Communication Bus Module. The ultraviolet detector was set to 235 nm. The samples were diluted in methanol (Merck)/1N HCl (90/10, vol./vol.) and 10 μL was injected through an XTerra RP C-18 column, 100 × 4.6 mm (Waters) set to a temperature of 35°C. The flow rate was constant at 1.5 mL/min. A linear gradient method was used: initially 9/1 (vol./vol.) of 0.1% wt./vol. NH_4_Ac (Sigma-Aldrich) in water/ACN to 0/10 (vol./vol.) 0.1% wt./vol. NH_4_Ac in water/ACN after 10 min. This was followed by a 5-min purge with 100% ACN. The limit of quantification and limit of detection values of this validated method are 0.22 and 0.065 μg/mL, respectively.

##### Ultra Performance Liquid Chromatography

Drug release following *in vitro* dissolution was quantified using an UPLC equipped with a ultraviolet detector (Waters, H-Class) set to 280 nm. The selected mobile phase was 3/7 (vol./vol.) ACN (Merck)/0.1% trifluoroacetic acid (Merck) run in isocratic mode. The 0.5-mL/min flow rate and 2-μL injection volume flowed through an Acquity UPLC^®^ BEH C-18, 50 × 2.1 mm column (Waters) set to a temperature of 35 ± 5°C during the 1.2-min run time. A reference solution (0.0556 mg/mL) of N,N-dimethylformamide (Sigma-Aldrich) and a diluted reference solution (0.00556 mg/mL) of FLU in 1/1 (vol./vol.) water/N,N-dimethylformamide were prepared in duplicate. The diluted reference solution 1 was injected *n* = 5 as the calibration standard. The diluted reference solution 2 was injected *n* = 2 as a control. An *n* = 2 injection of diluted reference solution 1 was performed after ≤12 injections and after the last injection as a control. Test samples were injected as *n* = 2. The reported results as percent dissolved were calculated based on the *n* = 5 injections from the first diluted RS (0.00556 mg/mL). Peaks were integrated using the Empower 2 and 3 software.

##### Gas Chromatography

Residual FA was determined using gas chromatography (GC 7890A, Agilent, Diegem, Belgium) coupled to a flame ionization detector. Chromatographic separation was achieved using a 0.18 mm × 20 m fused silica column (Rxi-624MS) of 1-μm film thickness. Injector and detector temperatures were set to 260°C and 270°C, respectively. A constant H_2_ gas flow was set to 1 mL/min. The oven program used was in 2 steps: (1) starting temperature 45°C held for 0.2 min, ramp 2°C/min up to 50°C, and (2) ramp 35°C/min to 250°C and held for 1.60 min (total run time of 10 min). Samples were prepared by transferring the content of 5 mg API in a 10-mL headspace vial and adding 2.5 mL dilution solvent containing 1/9 (vol./vol.) 1-propanol (Acros, Geel, Belgium)/dimethyl sulfoxide (Sigma-Aldrich, Diegem, Belgium) and 0.5-mL sulfuric acid (Merck). Concentration was determined based on a 1-point calibration curve on 5000 ppm.

DCM was analyzed by GC (EDMS-ERI-7845511:2.0, Agilent) coupled to a flame ionization detector. Chromatographic separation was achieved using a 0.32 mm × 50 m fused silica column, coated with chemically bonded polydimethylsiloxane phase (CP-SIL 5 CB) of 5-μm film thickness. Injector and detector temperatures were set to 230°C and 270°C, respectively. A constant N_2_ gas flow was set to 25 mL/min. The oven program used was in 2 steps: (1) starting temperature 40°C held for 0.5 min, ramp 5°C/min up to 170°C, (2) ramp 30°C/min to 220°C and held for 11.83 min. Samples were prepared by dissolving 25 mg of API in 2 mL of 1,3-dimethyl-2-imidazolinone (Sigma-Aldrich) in a 22-mL gas-tight vial. Concentration was determined based on a 3-point calibration curve ranging from 0.98 to 24.59 mg/mL.

##### Liquid Chromatography–Tandem Mass Spectrometry

Plasma samples were prepared by protein precipitation (ACN) and the supernatant was diluted with 0.1% FA in Milli-Q water/ACN (8/2 vol./vol.). The stable isotope labeled FLU-d3 (Sigma-Aldrich) was used as the internal standard. Samples were then analyzed by liquid chromatography–tandem mass spectrometry in the range of 5-5000 ng/mL. The system consisted of a Turbo-Ionspray™ interface (in positive mode) coupled with a Shimadzu liquid chromatogram (LC20AD, Brussels, Belgium). The LC system was equipped with a pump, oven, communication bus module, and degasser. The chromatographic separation was established on a 5 cm × 2.1-mm column, packed with 3.5 μm X-Bridge C18 (Waters) operating at 30°C. A gradient elution started at ACN/0.1% FA in Milli-Q water (50/50 vol./vol.) and completed at 98/2 vol./vol. The multiple reaction monitoring transitions were captured at 314.1-282 and 317.1-282.

#### N_2_ Physisorption

Nitrogen adsorption isotherms of all OMS materials were measured at −196°C using a Micrometrics Tristar II 3020-apparatus (Brussels, Belgium). Samples were pre-treated for 2 h at 30°C under a nitrogen flush. The pore volume and the surface area were calculated using the Brunauer-Emmett-Teller theory. The mesopore size distribution was derived from the desorption isotherm branch using the Barrett-Joyner-Halenda model.

### *In Vivo* Experiments

All *in vivo* studies were performed in accordance with the European Directive 2010/63 and Belgian laws regarding the protection of laboratory animals. Male Sprague-Dawley rats were given access to food and water *ad libitum*. An oral dose of 20 mg/kg was administered as either a suspension or capsule blend. Crystalline FLU was administered to 200-300 g rats as a micro- and nanosuspension. Microsuspensions containing 2 mg/mL FLU were prepared with 1 mg/mL docusate sodium (Cytec Industries BV, Vlaardigen, The Netherlands), 12 mg/mL Avicel^®^ RC591 (FMC International, Cork, Ireland), 20 mg/mL PVA 17 (BASF), 10 mg/mL Aqualon^®^ sodium carboxymethylcellulose (Ashland, Sint-Gillis-Waas, Belgium), 1.8 mg/mL methylparaben (Merck), and 0.2 mg/mL propylparaben (Merck). Nanosuspensions also containing 2 mg/mL FLU were prepared with 0.5 mg/mL Poloxamer 338 (BASF) and 0.05 mg/mL docusate sodium. Amorphous suspensions containing either standard spray-dried dispersion (SSDD), MPSDD or 3/7 (wt./wt.) FLU/OMS were prepared with 0.5-wt.% Methocel immediately prior to dosing. The OMS formulation was administered both as a suspension and capsule blend. The 4/6 (wt./wt.) FLU/OMS was the only formulation capable of fitting the target dose into one capsule. Moreover, it was postulated that the suspension vehicle would quickly enter the mesopores and push the API into the surrounding media, thus leading to rapid precipitation prior to dosing.^24^ Capsule blends were prepared according to the specific weight of each rat (325-355 g) following the blend ratios previously described (section 2.2.3) and placed into a size 9 elongated capsule (Torpac, Fairfield, NJ). Blood samples (60 μL) were collected from the lateral tail vein at *t* = 0.5, 1, 2, 4, 7, and 24 h after dosing. Samples were centrifuged at 1500 × *g* (Hettich, Tuttlingen, Germany) for roughly 10 min in ambient conditions within 1 h after sampling. Plasma samples after dosing were analyzed by liquid chromatography–tandem mass spectrometry. A non-compartmental pharmacokinetic (PK) analysis was performed using WinNonlin Professional (Version 5.2.1). Peak plasma concentrations (*C*_max_) and corresponding peak times (*T*_max_) were calculated. The area under the plasma concentration–time curve from time zero to time *t* (AUC_0-t_), where *t* is the sampling time corresponding to the last measurable concentration above the lower limit of quantification, and from time zero to infinity (AUC_0-inf_) were calculated using the linear up/log down trapezoidal rule.

## Results

**Figure 1:**
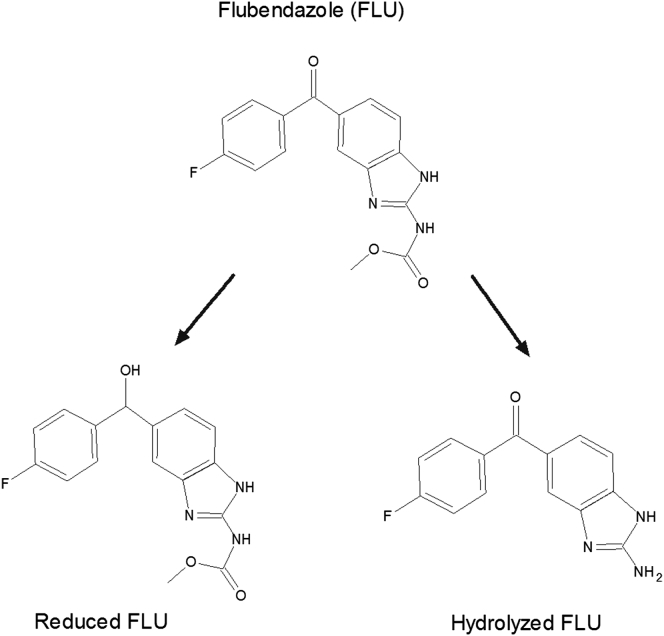
Structure of flubendazole (FLU) and its 2 metabolites.

The structure of FLU and its 2 metabolites, the hydrolyzed and reduced forms, are illustrated in Figure 1. The carbamate group is the main source of FLU’s extremely low aqueous solubility of 5 ng/mL (Table 1).

### Development Challenges With FA

As shown in Table 1, FLU also exhibits very low solubility in common organic solvents, but a very high solubility in FA, which ionizes the carbamate group (pKa 3.6) to increase the solubility. For successful employment of SDD and OMS technologies, a suitable concentration of dissolved API is required. Moreover, the selected solvents ideally exhibit a low vapor pressure for adequate removal following processing (SDD) or drug loading (OMS). Although a small amount of FA is sufficient to significantly improve the solubility, one major challenge is the elimination of residual FA due to its relatively high boiling point (∼101°C).^25^

#### OMS Drug Loading

**Figure 2:**
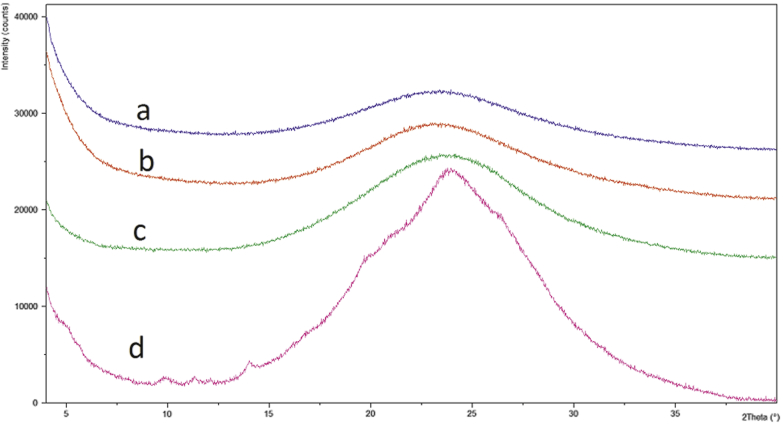
PXRD patterns (a) 2/8, (b) 3/7, (c) 4/6, and (d) 1/1 FLU/OMS (wt./wt.) following drug loading.

Due to its low volatility, DCM is a favorable solvent for OMS drug loading. However, the addition of FA was necessary in order to achieve a concentrated FLU solution necessary for drug loading (Table 1). A 1/1 (vol./vol.) FA/DCM solution was selected to load the 2/8 (wt./wt.) FLU/OMS. Pure FA was necessary to increase the drug load, as determined during early drug loading development studies. Figure 2 illustrates the PXRD patterns of 2/8, 3/7, 4/6, and 1/1 (wt./wt.) FLU/OMS. The presence of small Bragg peaks following 1/1 (wt./wt.) FLU/OMS drug loading corresponds to crystalline FLU. Assuming that mesopore-associated API is amorphous as a result of confinement effects, Bragg peaks in the sample suggest that some API is located outside of the mesopores.^26^ Therefore, no further investigations were conducted with the 1/1 (wt./wt.) FLU/OMS formulation.

**Figure 3:**
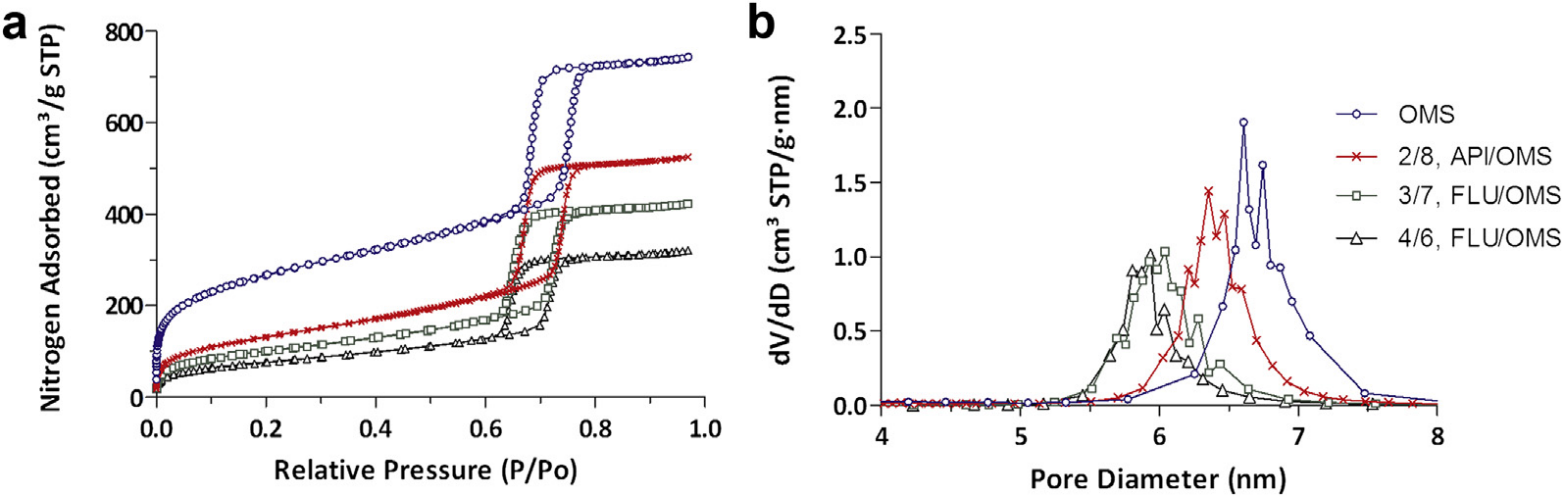
(a) Nitrogen adsorption/desorption isotherm and (b) Barrett-Joyner-Halenda determined pore diameter distribution of OMS before and after drug loading. All ratios expressed in weight.

The nitrogen physisorption results (Fig. 3) before and after drug loading reveal an overall decrease in available pore volume with an increase in drug load (Fig. 3a). A 740 cm^3^/g total volume was initially determined and decreased to approximately 350 cm^3^/g following 40% weight drug loading. Likewise, as seen in Figure 3b, the pore volume and pore size decreased as drug load increased, both of which indicate that the API is loaded inside the mesoporous structure.

#### SSDD Process

**Figure 4:**
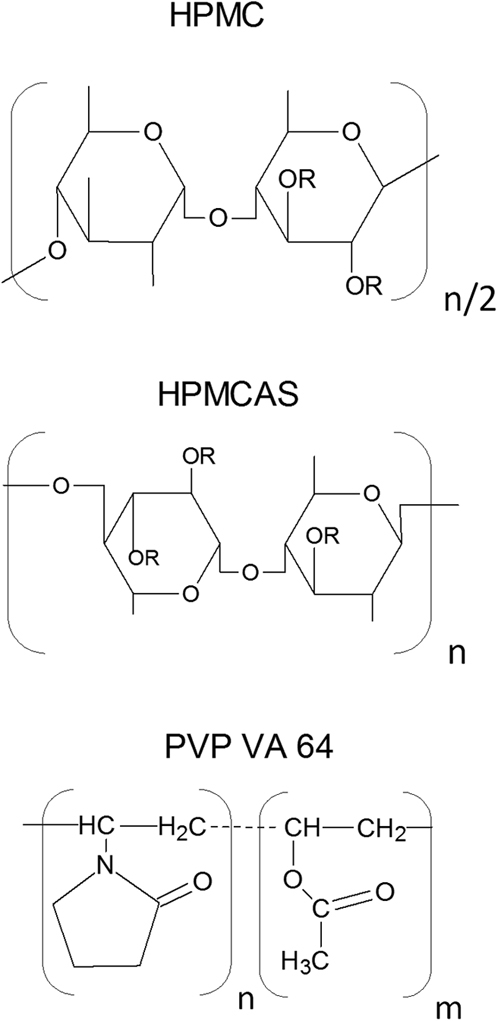
Chemical structure of polymers selected for SSDD and MPSDD (top) HPMC, where *R* is either hydroxyl, methoxyl, 2-hydroxypropoxyl; (middle) HPMCAS, where *R* represents either hydroxyl, methoxyl, 2-hydroxypropoxyl, acetyl, or succinoyl; (bottom) PVP VA64, where *n* = 1.2 m.

**Figure 5:**
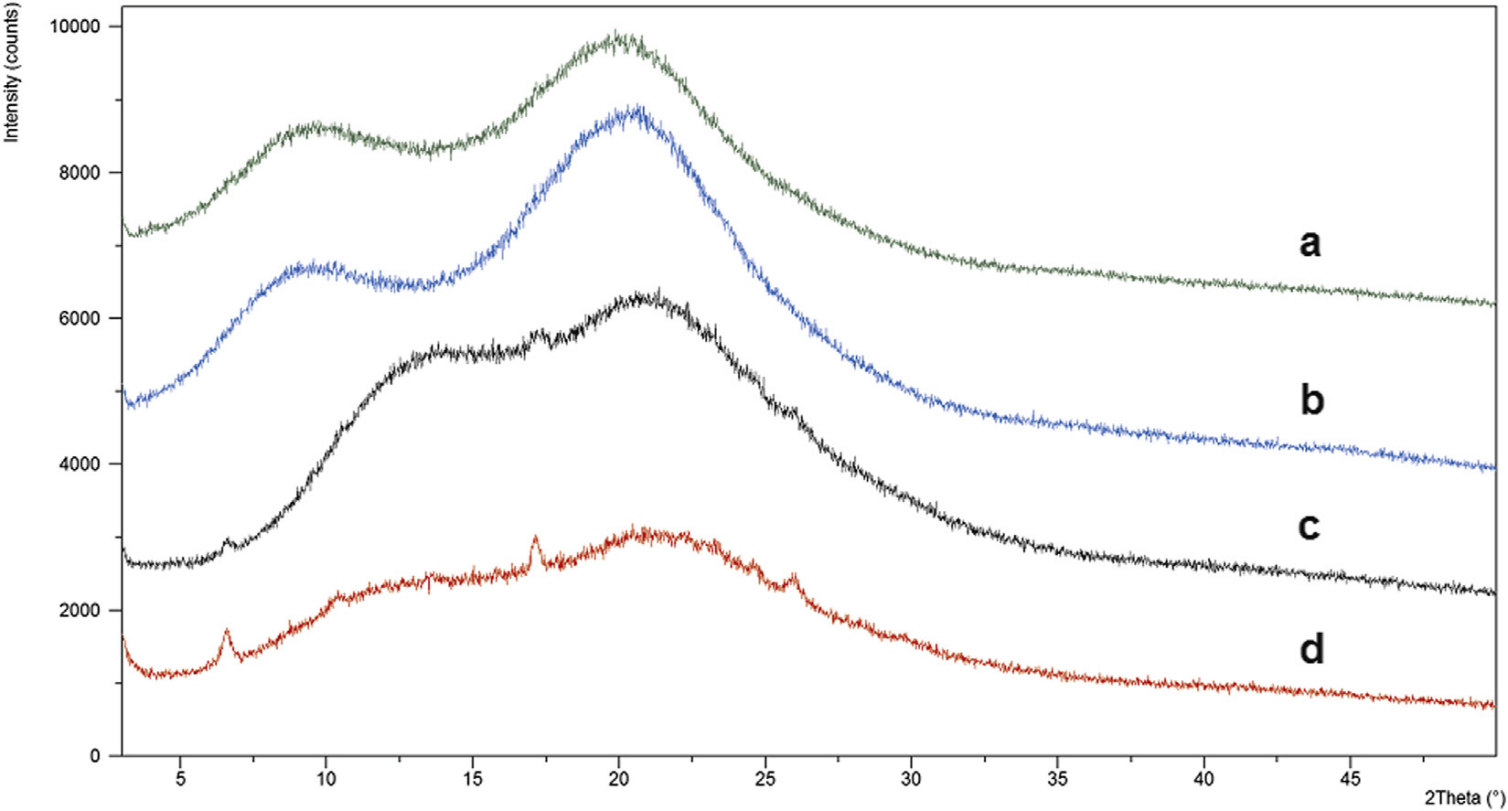
PXRD patterns of SSDD: FLU/HPMC E5/Vit. E TPGS: (a) 1/9/0.5 and (b) 1/3/0.15; FLU/PVP VA 64/Vit. E TPGS: (c) 1/9/0.5 and (d) 1/3/0.15 following post drying. All ratios expressed in weight.

As with the OMS formulation, FA was necessary to increase the solubility required for spray drying. Based on an internally developed spray dry model, a feedstock concentration of 1/9 (wt./wt.) FA/DCM was calculated as suitable for the equipment and working safety. A high and low API concentration of 1/9/0.5 or 1/3/0.15 FLU/polymer/Vit. E TPGS wt. ratio was selected to bracket the concentrations that were initially evaluated. Vit. E TPGS was selected as a precipitation inhibitor, based on automated screening experiments. The chemical structure of selected polymers is shown in Figure 4. PVP VA 64 was selected based on results from early developmental studies and HPMC E5 is a preferred polymer for spray dry experiments. Based on PXRD results, samples prepared with HPMC E5 and PVP VA 64 were amorphous and partially crystalline (Fig. 5). Based on these results, SSDD development was only continued with HPMC E5 samples.

#### Residual Solvents Following Secondary Drying

**Figure 6:**
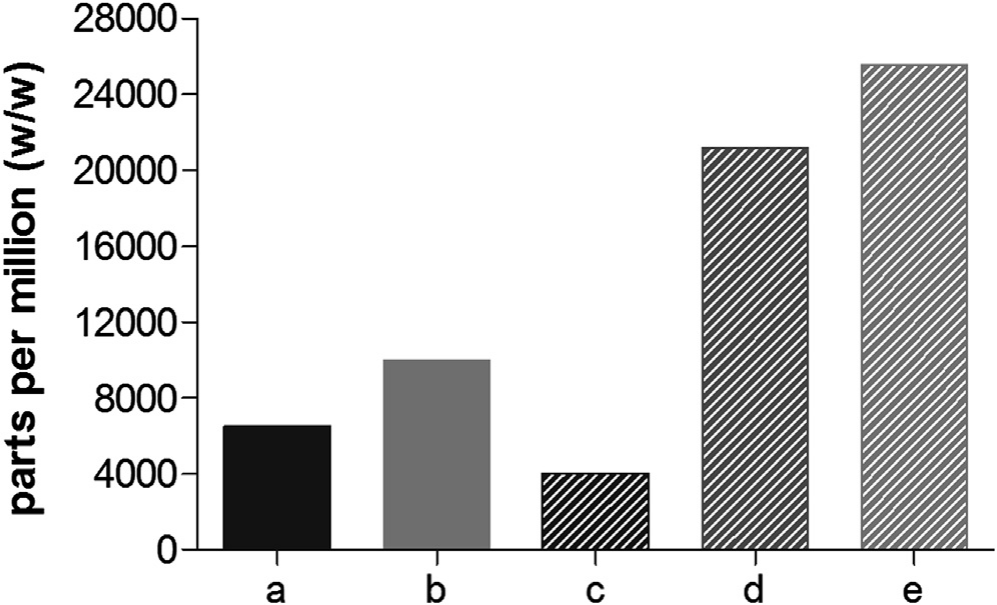
Residual FA following overnight post drying in SSDD, FLU/HPMC E5/Vit. E TPGS (a) 1/9/0.5, (b) 1/3/0.15 and FLU/OMS, (c) 2/8, (d) 3/7, and (e) 4/6. All ratios expressed in weight.

GC was used to determine the residual amount of FA and DCM in FLU-loaded OMS and SSDD. The detected DCM concentration was below the reporting threshold of 50 ppm in all formulations. The residual FA concentrations are shown in Figure 6. For the OMS concept, the FA concentration significantly increases with increasing drug load. The large increase from 4000 ppm (2/8 FLU/OMS) to 21,000 ppm (3/7 FLU/OMS) is attributed to the additional amount of FA required to increase the drug loading. To investigate the influence of drying time, SSDD 1/3/0.15, FLU/HPMC E5/Vit. E TPGS wt. ratio was measured following overnight and 5-day post drying. Here, no decrease in residual FA was observed following 5 days drying. There was, however, a small decrease (∼2%) in assay, as determined by HPLC (data not shown).

### Spray Dry Process Modifications

This approach uses a flash nozzle, rather than a pressure swirl or twin-fluid atomizers as with a standard spray dry operation. Here, atomization occurs via boiling of the solvent as it rapidly depressurizes to ambient conditions. Based on the Gibbs-Helmholtz equation (Eq. 1), the incorporation of heat increases the solubility of FLU in commonly used spray dry solvents and bypasses the need for FA. The resulting powder from these adaptations is described here as an MPSDD.

(1) $${\left(\frac{\partial G/T}{\partial T}\right)}_{p}=\;-\;\frac{H}{T^{2}}$$

**Figure 7:**
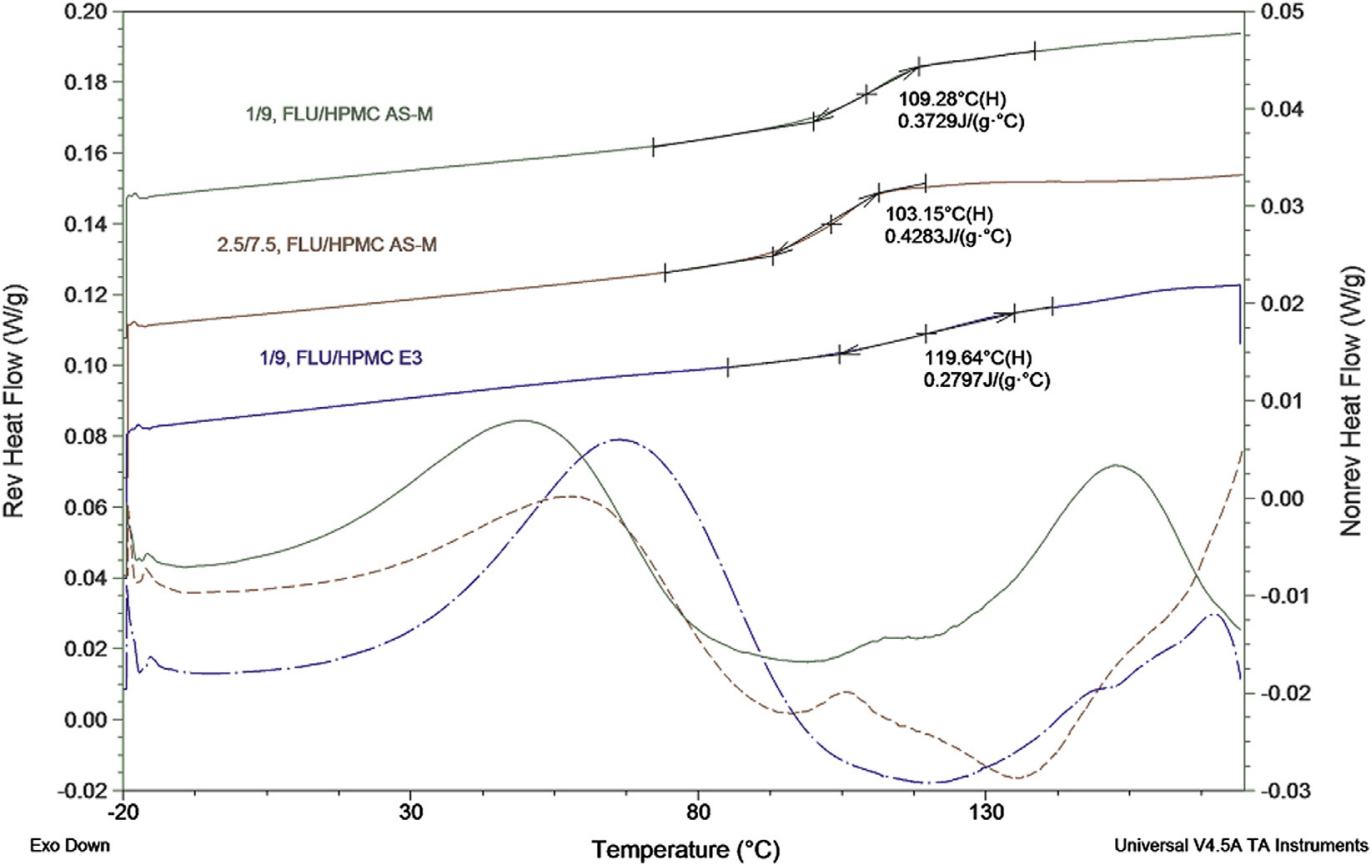
mDSC of MPSDD following manufacture.

Amorphicity of MPSDD was verified using PXRD (data not shown) and mDSC (Fig. 7). Formulations were equilibrated overnight (<5% RH) prior to mDSC measurements. The thermograms reveal a single *T*_g_, verifying a molecular dispersion. MPSDD prepared with HPMCAS-M resulted in a *T*_g_ of approximately 109°C and 103°C for 1/9 and 2.5/7.5 (wt./wt.) FLU/HPMCAS-M, respectively. MPSDD prepared with 1/9 (wt./wt.) FLU/HPMC E3 resulted in a slightly higher *T*_g_ of 120°C. Based on the slower release behavior from early development microcentrifuge dissolution tests (see Supplementary Data), the 2.5/7.5 (wt./wt.) FLU/HPMCAS-M was not further evaluated. In general, all the formulations dissolved in gastric medium to above the crystalline API solubility and sustained supersaturation of total solubilized drug species after a 1:1 transfer into simulated intestinal media. The 1/9 (wt./wt.) FLU/polymer MPSDD provided a higher concentration of total solubilized drug species than the 2.5/7.5 (wt./wt.) HPMCAS-M, and for this reason, not chosen for progression to *in vivo* studies.

**Figure 8:**
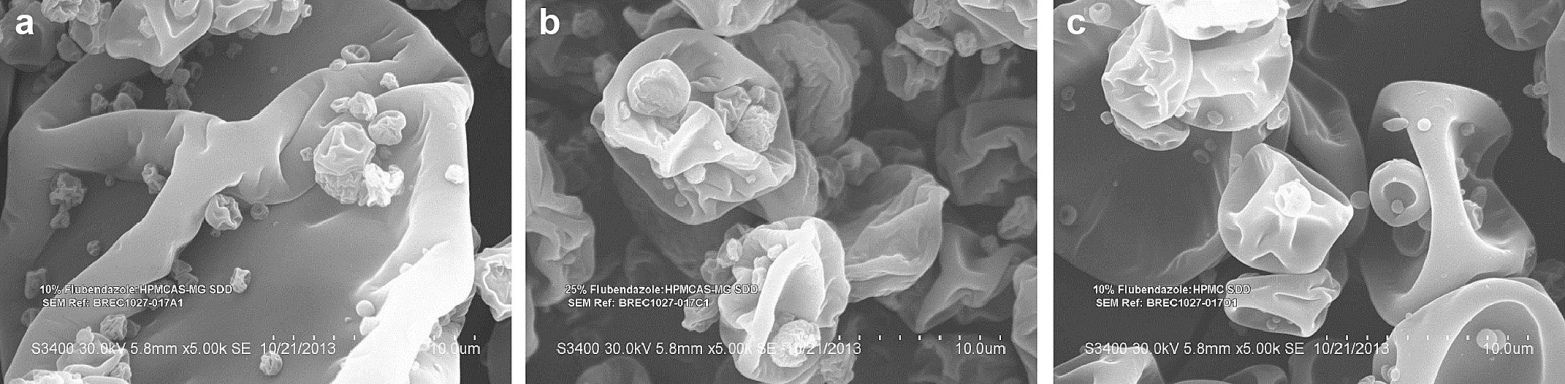
Scanning electron microscopy micrographs of MPSDD (a) 1/9 FLU/HPMCAS-M, (b) 2.5/7.5 FLU/HPMCAS-M, and (c) 1/9 FLU/HPMC E3. All ratios expressed in weight.

Scanning electron microscopy was used to investigate the resulting particle morphology of MPSDD powders. As pictured in Figure 8, they exhibit a spherical and corrugated shape, similar to that of a standard spray-dried dispersion with no appearance of crystalline API.

### Physicochemical Stability

The leading formulations from each amorphous concept were placed in open dish containers and exposed at 25°C/60% RH and 40°C/75% RH and analyzed at *t* = 0 and *t* = 2 weeks. These aggressive conditions were selected as a fast screening tool as part of the *in vitro* evaluations. PXRD was used to assess physical stability. HPLC assay and purity were used to determine chemical stability.

#### Physical Stability

**Figure 9:**
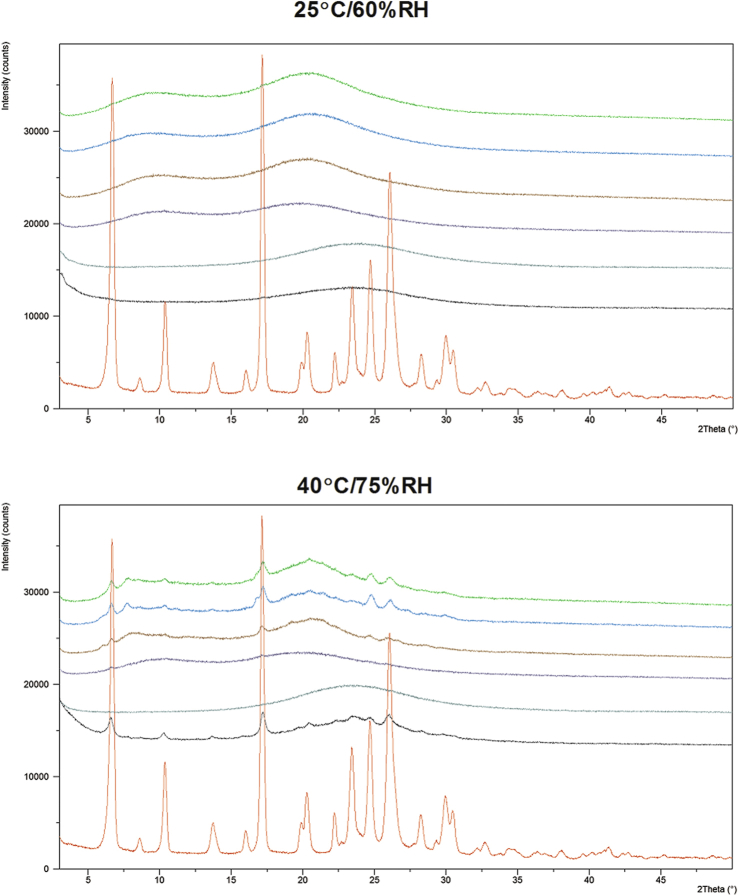
PXRD patterns of SSDD: 1/9/0.5, 1/3/0.15, FLU/HPMC E5/Vit. E TPGS; MPSDD: 1/9 FLU/HPMC E3, 1/9 FLU/HPMCAS-M; OMS: 3/7, 4/6, FLU/OMS following 2 weeks' exposure in open conditions and crystalline FLU. All ratios are expressed in weight and are listed from top to bottom in the diffraction profiles.

As shown in Figure 9, all formulations were stable at 25°C/60% RH for up to *t* = 2 weeks. However, both 1/3/0.15 and 1/9/0.5 (FLU/HPMC E5/Vit. E TPGS) SSDD formulations began to crystallize after 2 weeks' exposure at 40°C/75% RH. The 1/9 (FLU/HPMC E3) formulation produced by the MPSDD also showed signs of crystallization following 40°C/75% RH exposure. However, the 1/9 (wt./wt.) FLU/HPMCAS-M remained amorphous following storage in both conditions. These results indicate that the crystallization of the 1/9 (wt./wt.) FLU/HPMC E3 was due to the weaker API-polymer interaction rather than the modified spray dry process itself. One additional consideration for the crystallization that was observed for the HPMC E3 containing dispersion could be the effect of water uptake and resulting decrease in *T*_g_ at elevated humidity conditions.

Diffraction patterns of OMS formulations reveal that physical stability depends on the drug loading. The 4/6 (wt./wt.) FLU/OMS crystallized following 40°C/75% RH conditions, whereas the 3/7 (wt./wt.) FLU/OMS remained amorphous.

#### Chemical Stability

**Figure 10:**
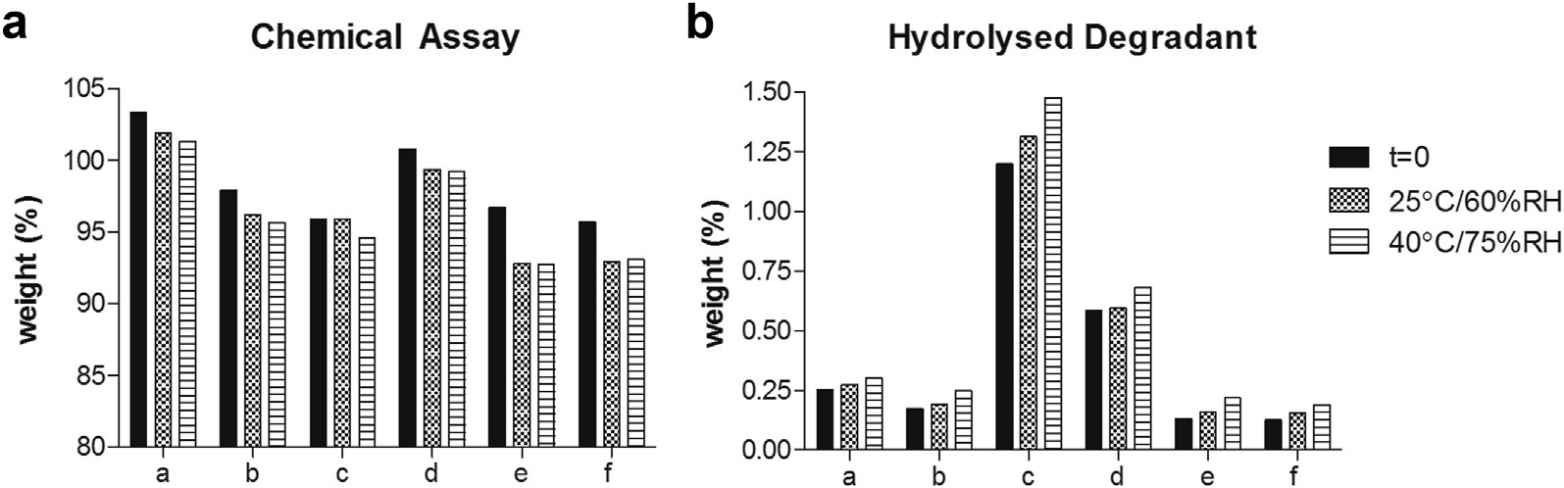
(a) Chemical assay and (b) hydrolyzed degradant as determined by HPLC following open conditions of SSDD: FLU/HPMC E5/Vit. E TPGS (a) 1/9/0.5, (b) 1/3/0.15; MPSDD: (c) 1/9, FLU/HPMCAS-M, (d) 1/9, FLU/HPMC E3; and FLU/OMS: (e) 3/7 and (f) 4/6. All ratios expressed in weight.

Figure 10a illustrates the chemical assay results. At *t* = 0, the assay values ranged from 95.7% (4/6 FLU/OMS) to 103.3% (1/9/0.5 FLU/HPMC E5/Vit. E TPGS). Following 2 weeks' exposure, this value decreases approximately 1%-2% wt. for SSDD and MPSDD formulations. The OMS-based concepts resulted in the largest decrease of around 4% wt.

Purity results revealed that the main degradation peak observed in all formulations was attributed to the hydrolyzed form. Figure 10b illustrates the concentration of this hydrolyzed degradant in all tested samples. MPSDD formulations resulted in the highest initial hydrolyzed concentration. The 1/9 (wt./wt.) FLU/HPMCAS-M resulted in over twice the concentration to that of 1/9 (wt./wt.) FLU/HPMC E3. The OMS concepts resulted in the overall lowest hydrolyzed concentration with no significant difference observed between 3/7 and 4/6 (wt./wt.) FLU/OMS. Formulations from all amorphous concepts resulted in an increasing concentration with increased stressed conditions. At *t* = 0, the total degradation products of HPMCAS-M was highest at 2.12 and lowest for 4/6 (wt./wt.) FLU/OMS at 0.66.

### *In Vitro* Release Behavior

**Figure 11:**
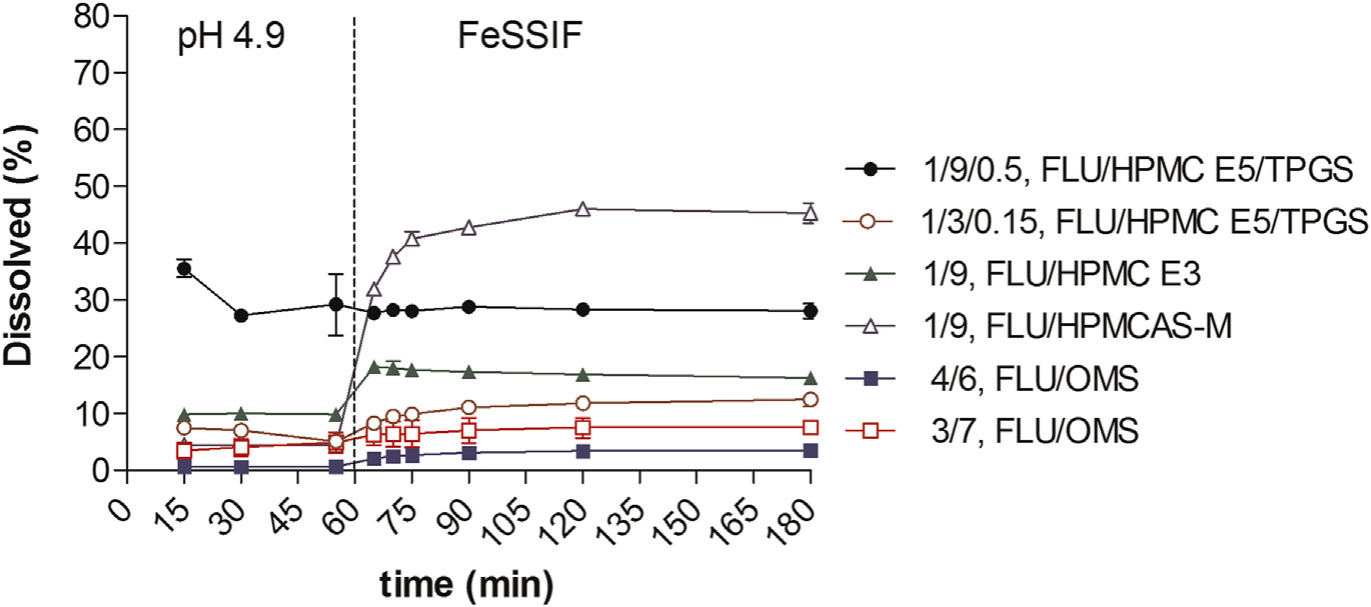
Dissolution profiles of 2 mg/mL suspensions in 0.5%-wt. methocel and 4/6 FLU/OMS capsule blend. Formulations adjusted to 50 mg of API per dissolution vessel. Points represent the average of *n* = 2 and bars reflect the range. All formulation ratios expressed in weight.

The *in vitro* release behavior of leading formulations was evaluated in bi-phasic biorelevant media to simulate the human fed stomach into the fed intestine situation (Fig. 11). Both OMS formulations resulted in the lowest overall release behavior in pH 4.9 and FeSSIF. The slightly higher release rate from the OMS suspension could be attributed to the faster ingress of media into the pores and therefore faster release into the surrounding media. The SSDD formulation containing 1/9/0.5 FLU/HPMC E5/Vit. E TPGS wt. ratio maintained an overall similar release profile in pH 4.9 and FeSSIF of roughly 28%-35% dissolved. HPMCAS-M is a pH-dependent polymer that begins to solubilize at pH >4.5. This is reflected in the release behavior of MPSDD: 1/9 FLU/HPMCAS-M. An initial % dissolved of roughly 4% is observed in pH 4.9 then rapidly increases to 45% in FeSSIF, resulting in the overall highest amount of % dissolved compared to all other formulations. However, the MPSDD 1/9 (wt./wt.) FLU/HPMC E3 performed slightly better than the SSDD 1/3/0.15, FLU/HPMC E5/Vit. E TPGS wt. ratio. Both OMS formulations resulted in the overall lowest % dissolved in pH 4.9 and FeSSIF. Poor wettability was visually observed with all formulation concepts (i.e., poorly wetted powder remaining in the syringe) and with the OMS capsule blend (i.e., particles sticking to the sinker).

### Bioavailability in Rats

**Figure 12:**
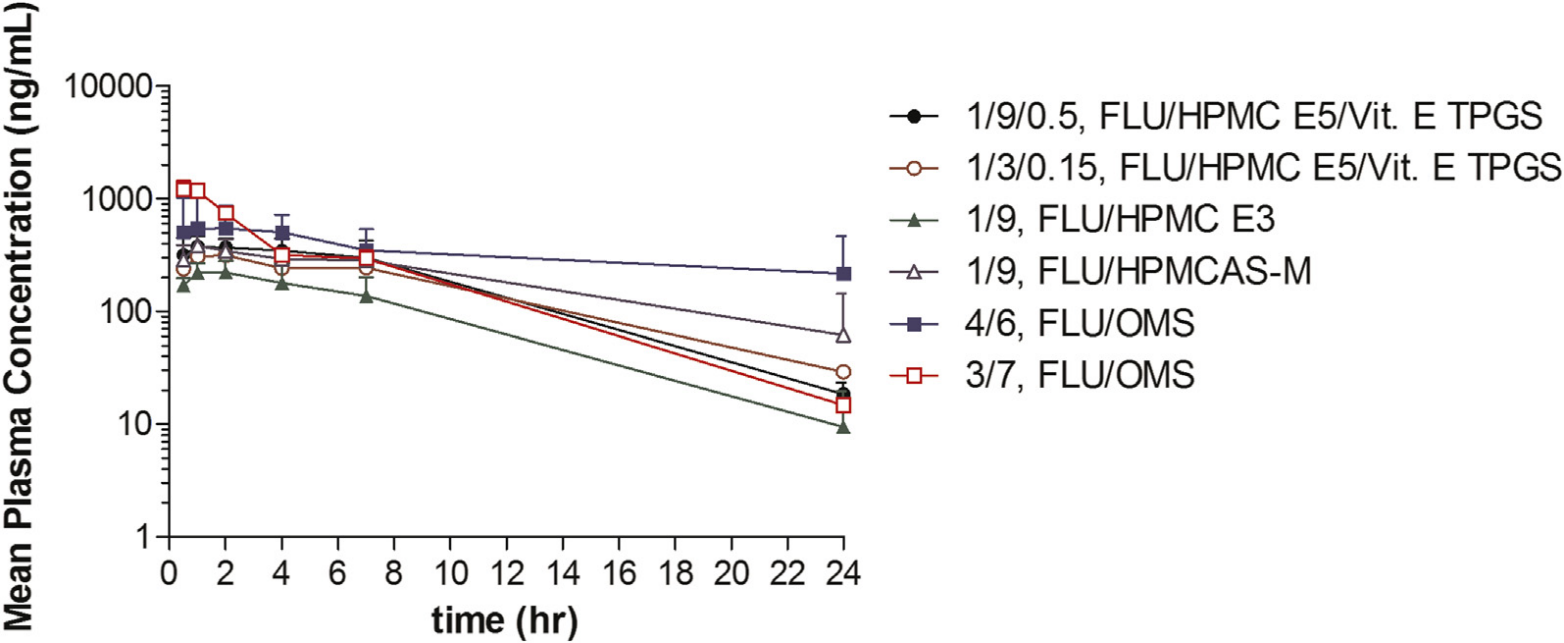
Plasma concentration–time profile of FLU following oral administration (20 mg/kg) to male Sprague-Dawley rats. Points represent the mean values of *n* = 3 ± standard deviation for suspensions and *n* = 4 ± standard deviation for 4/6 (wt./wt.) FLU/OMS capsule blend. All formulation ratios expressed in weight.

The plasma concentration–time profiles of the FLU compound are provided in Figure 12. As with the dissolution experiments, poor wettability was again observed when preparing the suspensions immediately prior to dosing. All amorphous concepts formulated with polymer (SSDD and MPSDD) resulted in similar *T*_max_ values of 1.3-1.7 h (Table 2). The 3/7 (wt./wt.) FLU/OMS suspension resulted in the fastest *T*_max_ of 0.8 h and highest *C*_max_ value of 1220 ng/mL. The 4/6 (wt./wt.) FLU/OMS capsule blend plasma profile was distinct from all the other concepts. Although this formulation had a later *T*_max_ value of 2.4 h, it resulted in the overall highest AUC_0-24 h_ of 7800 ng·h/mL. Both crystalline suspensions resulted in the slowest *T*_max_ value of 3.0 h with the microsuspension exhibiting the overall lowest *C*_max_ value of 102 ng/mL. Compared to the microsuspension, the *C*_max_ and AUC_0-7 h_ value of the nanosuspension increased 3-fold to 331 ng/mL and 1811 ng·h/mL, respectively.

## Discussion

The objective of this study was to formulate amorphous FLU to increase the dissolution rate and aqueous solubility to improve the systemic exposure. A 20-mg/kg dose was selected to compare the *in vivo* behavior between the different formulations, as this is a well-accepted dose level in the rodents to explore the PK. This compound was first developed for local activity against worms in the gastrointestinal tract and therefore exploring the plasma levels required for systemic exposure is still ongoing. Similarly, the relevant dose in man for efficacy and the desired shape of PK profile is under investigation.

Following oral administration in male rats, both crystalline suspensions resulted in the latest *T*_max_ of 3.0 h with the microsuspension exhibiting the overall lowest *C*_max_ of 102 ng/mL. Decreasing the particle size for the nanosuspension improved the *C*_max_ and AUC values by approximately 3-fold but these results were still not sufficient to increase the extent of supersaturation to improve absorption. The 3/7 (wt./wt.) FLU/OMS suspension exhibited the highest *C*_max_ value, followed by a steady decline in plasma concentration. This is due to its higher degree of supersaturation upon release, coupled with no precipitation inhibitors. Therefore, the compound is rapidly absorbed. The PK profile of the 4/6 (wt./wt.) FLU/OMS capsule blend resulted in the overall highest AUC value of approximately 7800 ng·h/mL but a later *T*_max_ of 2.4 h. This difference in *in vivo* behavior is likely due to the difference in drug release kinetics from the capsule blend formulation compared to the suspension and unlikely due to the small difference in drug load. In the case of the capsule, time is required for capsule disintegration and sodium lauryl sulfate in the blend will contribute to increasing the extent of supersaturation. Both of these will influence the shape of the plasma concentration time profile (Fig. 12).

*In vitro* dissolution was used as a tool to predict *in vivo* performance. The biorelevant media was selected to simulate the fed state in human. However, the *in vitro* and *in vivo* drug release profiles did not correlate well with each other. These observed differences could be attributed to a number of reasons. First, *in vitro* conditions do not account for any gastrointestinal tract metabolism and hydrodynamics that occur *in vivo*. Also, the biorelevant media selected (pH 4.9-FeSSIF) does not accurately reflect the stomach and intestinal tract pH conditions in rat, especially for pH-sensitive polymers (i.e., HPMCAS-M).^27^

During the microcentrifuge dissolution test (Supplementary Data), it was observed that the MPSDDs had a range of improvement from 2 to 20 times that of the crystalline API, according to the AUC. Based on the observed enhancement in the microcentrifuge dissolution test, it is expected that these formulations would have a range of enhancement when evaluated in the rodent. In general, formulations are expected to have enhanced bioavailability relative to the crystalline API.

Due to its high *T*_m_ of 238°C (Table 1) and observed degradation upon melting, a drug delivery technology that destroys the crystal lattice by virtue of heating (i.e., hot melt extrusion) was not feasible. A high amorphous drug load of <40% wt. with OMS containing a pore size of approximately 6.6 nm (Fig. 3) was achieved due to its high pore volume (∼1 mL/g) and extremely large specific surface area (∼925 m^2^/g). These unique characteristics allow for high drug loading capacity and potential for drug adsorption.^28^, ^29^

Based on Flory-Huggins solution theory, miscibility and phase behavior of SDDs is influenced by the API-polymer interaction.^30^ In the case of SSDD, an amorphous solid dispersion was achieved with HPMC E5 but not with PVP VA64, a co-polymer manufactured from vinylpyrrolidone and vinylacetate monomers in blocks of 6 and 4 monomers, respectively (Fig. 4).^31^ This difference in ability to interact between FLU and both polymers might be at the base of the difference in ability to form an amorphous solid dispersion. Another reason could be the difference in *T*_g_ between both polymers, as HPMC has a higher *T*_g_ of ∼145°C compared to that of PVP VA64 of ∼107°C.^32^, ^33^, ^34^ In contrast to PVP VA64, the ability for FLU to form an amorphous solid dispersion with HPMC E5 suggests that the use of a higher *T*_g_ excipient could make the API less prone to recrystallization.

The use of FA to dissolve the API was also necessary with the SSDD and OMS concepts. Although FA is placed in the safest category of class III residual solvents, based on toxicity and degree of environmental hazard, it still invites additional downstream process challenge and considerations.^35^ The SSDD concepts were produced in experimental scale equipment where the main components are glass. Should this process need to be scaled up, the 1/9 (wt./wt.) FA/DCM solvent system may be incompatible with the stainless steel metal components in commercial-scale spray dryers. Similarly, careful attention to the equipment is required when drug loading the OMS with both the 1/1 (vol./vol.) FA/DCM and pure FA solutions.

Another downstream process concern is the removal of FA. The International Conference of Harmonisation guidance for industry, Q3C Impurities: Residual Solvent (ICS Q3C), limits FA to 5000 ppm and a permitted daily exposure of 50 mg/day. As shown in Figure 6, the 3/7 and 4/6 (wt./wt.) FLU/OMS formulations are over 5-fold the 5000 ppm limit. Although no formal drying studies were conducted, it is unlikely that the residual FA in these OMS formulations can be reduced significantly when performing drug loading with pure FA. The effect of drying time of 5 days in 45°C and 200 mbar under nitrogen flow was investigated using one SSDD formulation with no change in residual FA, albeit a small increase in chemical degradation (data not shown). Based on results in Figure 10 and FLU’s tendency to degrade upon heating, it is hypothesized that increasing the drying temperature from 45°C will also decrease the chemical purity.

The use of heat during processing (MPSDD) circumvents the need for FA, but resulted in a decrease in chemical purity. However, the additional heat step is not the sole contributing factor to this. The 1/9 (wt./wt.) FLU/HPMCAS-M resulted in over twice the amount of hydrolyzed degradant compared to 1/9 (wt./wt.) FLU/HPMC E3 (Fig. 10b). This is likely the result of HPMCAS-M acetate succinate group interaction with the FLU’s hydrolysis-sensitive carbamate group (Figs. 1 and 4). Although the OMS concept resulted in highest chemical purity, it did result in the largest decrease in assay following storage (Fig. 10a).

Although the 1/9 (wt./wt.) FLU/HPMCAS-M resulted in the largest decrease in chemical purity, it was one of the most physically stable formulations following 40°C/75% RH, together with 3/7 (wt./wt.) FLU/OMS. All formulations remained amorphous following storage for 2 weeks at 25°C/60% RH. During preliminary evaluations of the MPSDD technology, the *T*_g_ following overnight equilibration at <5%, 50%, and 75% RH was determined (data not shown). As illustrated in Figure 7, the *T*_g_ of MPSDD prepared with HPMC E3 is ∼120°C (<5% RH) but decreased to 60°C following 75% RH exposure. In the case of HPMCAS-M, the *T*_g_ equilibrated at <5% RH is ∼110°C (Fig. 7) and decreased to 70°C following 75% RH conditions. These results show that HPMCAS-M takes up less water than HPMC and is more effective in reducing the API’s molecular mobility. Therefore, differences in moisture uptake can explain the crystallization of 1/9 (wt./wt.) FLU/HPMC in 40°C/75% RH, whereas 1/9 (wt./wt.) FLU/HPMCAS-M remained amorphous. The reason that the 4/6 (wt./wt.) FLU/OMS showed signs of crystallization while the 3/7 (wt./wt.) FLU/OMS did not is due to their difference in drug load. Upon drug loading, the API is pulled into the pores through capillary forces.^20^ Due to the silanol groups along OMS walls, the drug’s tendency is to attach to the walls, rather than accumulate in the center.^26^ Therefore, as the drug load increases, the API begins to distribute toward the open end of the pores, thus making it more susceptible to the external environment. The stability assessment in open conditions exposed FLU to harsh external conditions not representative of a finished and packaged product. Although the majority of formulations showed signs of crystallinity following *t* = 2 weeks at 40°C/75% RH, this does not necessarily mean that they are not developable. These aggressive conditions were selected as part of our evaluation for selecting the most robust amorphous formulation.

## Conclusions

Results from this study exhibit the process challenges in not only formulating amorphous FLU but also stabilizing it. Formulations from 3 different drug delivery technologies were explored and optimized to improve the dissolution rate and extent of oral absorption. Physicochemical stability and *in vitro* release in biorelevant media were used to screen all formulations. The 2 emerging drug delivery technologies, an MPSDD 1/9 (wt./wt.) FLU/HPMCAS-M, and 3/7 (wt./wt.) FLU/OMS resulted in superior physical stability compared to the more conventional SSDD approach. Although the OMS technology resulted in the best chemical purity, the use of FA results in high amounts of residual solvent and complicates downstream processability. The heat step during MPSDD increased the amount of hydrolyzed degradant; however, the results showed that this was also dependent on the API-polymer interaction. Plasma concentration profiles from OMS formulations differed due to the mode of administration between the suspension and capsule blend. The 30% wt. loaded suspension resulted in the fastest *T*_max_, highest *C*_max_, and exhibited an overall distinctive profile from the other concepts. The 40% wt. loaded capsule blend resulted in the slowest *T*_max_ and highest AUC. Although the OMS illustrated promising *in vivo* performance, the MPSDD technique will be further explored based on the *in vitro* data and ease of manufacturing scalability.

## Figures and Tables

**Table 1:** Physicochemical Properties of Flubendazole (FLU)

Characteristic	Value
Molecular weight (g/mol)	313.1
*T*_m_ (°C)	238
*T*_g_ (°C)	156
pKa	3.6 and 9.6
Log*P*	3
*P*_app_ (10 μM)	AB: 33.8 × 10^−6^ cm/s
Solubility (mg/mL) in ambient conditions	
Water	0.005
Methanol	0.11
Acetone	0.18
Dichloromethane	0.14
Formic acid	340

**Table 2:** PK Parameters of FLU Following a 20-mg/kg Dose

Formulation	*C*_max_ (ng/mL)	*T*_max_ (h)	AUC_0-7 h_ (ng·h/mL)	AUC0_0-24 h_ (ng·h/mL)	AUC_0-∞_ (ng·h/mL)
FLU microsuspension	102 ± 14.1	3.0 ± 1.73	575 ± 60.5	NC	NC
FLU nanosuspension	331 ± 50.3	2.7 ± 1.15	1811 ± 347	2710 ± 347	3191 ± 690
1/9/0.5 FLU/HPMC E5/Vit. E TPGS	380 ± 89.7	1.3 ± 0.58	2300 ± 687	4030 ± 1320	4140 ± 1340
1/3/0.15 FLU/HPMC E5/Vit. E TPGS	320 ± 89.0	1.7 ± 0.58	1790 ± 489	3510 ± 952	3750 ± 990
1/9 FLU/HPMC E3	224 ± 51.3	1.7 ± 0.58	1230 ± 448	1940 ± 1110	2650 ± 562
1/9 FLU/HPMCAS-M	389 ± 97.3	1.3 ± 0.58	2110 ± 408	4320 ± 760	NC
4/6 FLU/OMS (capsule)	690 ± 359	2.4 ± 1.9	3240 ± 1820	7800 ± 5680	NC
3/7 FLU/OMS (suspension)	1220 ± 232	0.83 ± 0.29	3760 ± 317	5360 ± 287	5440 ± 290

Values represent the mean values of *n* = 3 ± standard deviation for suspensions and *n* = 4 ± standard deviation for 4/6 (wt./wt.) FLU/OMS capsule blend. All formulation ratios expressed in weight. AUC extrapolation exceeds 25%.

NC, not calculated.
