# Supplementary material for: Evaluation of Three Amorphous Drug Delivery Technologies to Improve the Oral Absorption of Flubendazole
Source: J Pharm Sci. 2016 Sep;105(9):2782–93. doi: 10.1016/j.xphs.2016.03.003 (PMC4988473; doi:10.1016/j.xphs.2016.03.003)
Supplement: Supplementary Data [file mmc1.docx]

MPSDD microdissolution test results. Points represent the average of *n* = 2 and bars reflect the range. All formulation ratios expressed in weight.
